# Threatened Birds in a Changing Mediterranean Wetland: Long-Term Trends and Climate-Driven Threats

**DOI:** 10.3390/life15060892

**Published:** 2025-05-31

**Authors:** Imane Bouregbi, Zinette Bensakhri, Rabah Zebsa, Abdelheq Zouaimia, Soufyane Bensouilah, Oualid Bouteraa, Rassim Khelifa, Mohamed Laid Ouakid, Hayat Mahdjoub, Moussa Houhamdi

**Affiliations:** 1 Department of Biology, Faculty of Sciences, University Badji Mokhtar, B.P. 12, Sidi-Ammar, Annaba 23000, Algeria; bouregbi.imane@gmail.com (I.B.); ouakidmomo@outlook.fr (M.L.O.); 2 Laboratoire Biologie, Eau & Environnement (LBEE), Faculty of SNV-STU, University of 8 May 1945 Guelma BP 4010, Guelma 24000, Algeria; bensakhri.zinette@univ-guelma.dz (Z.B.); zouaimia.abdelheq@gmail.com (A.Z.); houhamdi.moussa@univ-guelma.dz (M.H.); 3 Department of Biology, Faculty of Sciences, Amar Telidji Laghouat University, Laghouat 03000, Algeria; soufyaneben@hotmail.com; 4 Laboratory of Geology and Environment (LGE), University of Constantine 1, Constantine 025000, Algeria; oualid.bouteraa25@gmail.com; 5 Biology Department, Concordia University, 7141 Sherbrooke St. W., Montreal, QC H4B 1R6, Canada; rassim.khelifa@concordia.ca (R.K.); hayatmahdjoub@gmail.com (H.M.)

**Keywords:** abundance, drought, waterbirds, protected areas, breeding, wintering, population fluctuations, sustainable conservation, climate adaptation

## Abstract

Understanding the impact of climate change on waterbirds, particularly those of conservation concern, is essential for their long-term management and effective conservation. In arid regions such as North Africa, wetlands of international importance have been affected by severe drought events, but their impact on waterbirds is still not well understood. Here, we assess the population dynamics of four emblematic resident species (*Aythya nyroca*, *Marmaronetta angustirostris*, *Oxyura leucocephala*, and *Porphyrio porphyrio porphyrio*) in a North African Ramsar site (Garaet Hadj Tahar marsh, Northeast Algeria), and determine the climatic variables (temperature, precipitation, and drought index) that best predict the change in abundance in wintering and breeding seasons. We used a long-term survey of regular counts during the wintering and breeding seasons of 2002–2019. The temporal trend of waterbird abundance differed between species and seasons. Species showed different sensitivities to different climatic variables at different time windows preceding the wintering and breeding seasons. We found that the population dynamics of the endangered *O. leucocephala* in the wintering and breeding seasons were best predicted with the drought index, whereas those of *P. p. porphyrio* were best correlated with maximum temperature. Population dynamics during the wintering and breeding season of the two other species were best explained with either maximum temperature, precipitation, average temperature, or drought. Species responded differently to warming and drought. The most endangered species (*O. leucocephala*) showed population declines in drier years, whereas less threatened species (*A. nyroca*, *M. angustirostris*, and *P. p. porphyrio*) exhibited either positive or negative correlations. The observed temporal increase in vegetation cover was positively correlated with the wintering population size of *O. leucocephala*, as well as the breeding population size of *P. p. porphyrio* and *M. angustirostris*. These findings highlight the urgent need for climate-adaptive conservation strategies to sustain wetland biodiversity and ecosystem resilience in the face of increasing climate stressors, aligning with the global sustainability goals for biodiversity conservation and ecosystem management. Our results suggest that future increases in temperatures and drought severity might threaten endangered waterbirds and benefit more common species in the region.

## 1. Introduction

Wetlands are cornerstones of biodiversity, offering critical ecosystem services that greatly benefit nature and people [1,2]. A wide range of aquatic and terrestrial species depend on wetlands for various biological needs (e.g., drinking, feeding, resting, or mating), making them exceptional systems for ecological functioning across landscapes [3]. However, wetlands have been experiencing various types of climatic and anthropogenic stressors, changing their physical, chemical, and biological characteristics, and threatening the ecosystem services they provide (e.g., water regulation, nutrient cycling, carbon sequestration, and reservoir of biodiversity) [4,5]. For instance, the size of wetlands is projected to decline with climate change [6], and the human disturbance level is predicted to increase with human population growth and urban sprawling [7]. An effective conservation of wetlands requires an understanding of the contribution of different stressors on key bioindicators of ecosystem health [4].

Waterbirds are commonly used to monitor the ecological state of wetlands because their presence, abundance, and diversity reflect the quality of the habitat and the availability of resources [8]. They live in complex communities, occupying various trophic levels, and exploiting different habitat types that sustain a broad range of aquatic fauna. Thus, waterbirds are not only bioindicators, but they can also serve as umbrella species, benefiting a plethora of aquatic taxa [9]. However, waterbirds have been facing many threats worldwide [10], including climate change [11], habitat loss [12,13], and illegal hunting [14,15]. Although waterbirds might benefit from habitat protection [16], such protection might not be enough for the conservation of waterbirds under scenarios of severe climate change and anthropogenic disturbance [17]. The rapid response of waterbirds to climate change (e.g., change in population size, range shift, phenological shift) makes them great sentinels of how global changes impact biodiversity [18]. Effective conservation strategies and management practices require long-term data, but such data are not available in many areas that have been affected by climate change [19]. Waterbirds represent a notable exception, as they are among the few taxa for which comprehensive long-term datasets are available across various global regions.

Waterbirds in Mediterranean climates are under severe climatic stress, particularly because the increase in the intensity and duration of dry periods negatively influences the demographic parameters of populations [20]. In North Africa, most wetlands of international importance (Ramsar sites) are within the Mediterranean climate and most threatened species live in the hottest part of their geographic range (southern range) [21]. In Algeria, there are four resident species that have attracted considerable conservation and research attention during the last two decades for their global importance [21,22]. The white-headed duck (*Oxyura leucocephala*) is listed as “Endangered” in the IUCN Red List. This waterbird experienced a swift decline since the initiation of systematic winter counts in the 1960s, as revealed by long-term monitoring data [23]; however, the North African population can be considered relatively stable [22]. Marbled teal (*Marmaronetta angustirostris*) was recently downgraded from Vulnerable (1994–2017) to Near Threatened, but it is listed as Critically Endangered in some Mediterranean regions [24,25]. Ferruginous duck (*Aythya nyroca*) is presently assessed as “Near Threatened” due to the moderate declining pattern in population size. The global assessment of the latter two species has been challenging given the marked year-to-year fluctuation in range size and the temporal fluctuations of stable populations [26,27]. Purple swamp-hen (*Porphyrio porphyrio porphyrio*) has a widespread distribution and is evaluated as “Least Concern”. However, the overall population trend is unclear, and the global population size is still unknown [22]. The species is distributed in relatively small, isolated populations across numerous Algerian wetlands. This fragmented distribution likely increases its vulnerability to anthropogenic pressures, such as habitat loss, pollution, and human disturbances, which can have a disproportionate impact on smaller, less connected populations [22,28]. Most studies on these species have been conducted over short periods and have not assessed the impact of climate change on their population fluctuations. These four species were selected due to their conservation significance, ecological roles, and their sensitivity to climate change. The white-headed duck and marbled teal are highly specialized waterbirds dependent on permanent, high-quality wetlands, making them excellent indicators of wetland hydrology and long-term climate variability [29,30,31]. The ferruginous duck, which inhabits both permanent and temporary wetlands, represents species affected by habitat fluctuations driven by precipitation changes [32]. The purple swamp-hen, with its widespread but fragmented distribution, serves as a model for species with limited connectivity between wetland patches, making it a good indicator of habitat fragmentation effects [33]. Given their varied ecological requirements and responses to environmental changes, these species collectively represent different facets of wetland ecosystem functioning. Their population trends, distribution shifts, and demographic responses to climate stressors provide insights into how climate change alters wetland hydrology, resource availability, and community dynamics, thus making them useful bioindicators of broader ecosystem changes. However, to our knowledge, there are no published studies based on long-term observations in key wetlands of conservation importance in the region. Such studies would provide valuable insights into the impact of climate change on waterbirds and wetlands and inform decision makers in establishing effective mitigation measures.

In this study, we investigate how climate change influences the population dynamics of four threatened waterbird species (*Aythya nyroca*, *Oxyura leucocephala*, *Marmaronetta angustirostris*, and *Porphyrio porphyrio porphyrio*) through a long-term survey (2002–2019) in a North African Ramsar site (Garaet Hadj Tahar marsh, Northeast Algeria). We first performed two temporal assessments: we explored (1) how climatic conditions (temperature average and extremes, annual precipitation, and drought index [SPEI]) and vegetation (vegetation cover from satellite imagery) changed during the two decades; (2) how bird population sizes changed over time. For each of these assessments, we explored the temporal changes for the wintering and breeding seasons to understand the seasonal patterns of environmental and biotic changes. To understand which period of the year is the most correlated with bird abundance, we used a species-specific time-window approach that compares the magnitude of correlations across windows that differ in duration and position within the year. After selecting the time window, we tested for the additive effect of climate and vegetation on bird counts.

## 2. Materials and Methods

### 2.1. Study Area

The study was carried out in Garaet Hadj Tahar (36°51′50″ N, 07°15′57″ E); a 75 ha marsh in Skikda, northeastern Algeria. This marsh, which is a component of the Guerbes–Sanhadja complex, was designated as a Ramsar site on 2 February 2001, due to its geomorphological significance, as well as its function as a bioclimatic crossroads that promotes a high degree of biodiversity [34]. The complex comprises saline lagoons, including Garaet Dahria, freshwater lakes like Garaet Sidi Makhlouf, and freshwater marshes. It is situated along the eastern coast of Algeria in the wilaya of Skikda. The western borders of the region are the coastal highlands of Skikda, while the eastern borders are the coastal massif of Chetaïbi. The water depth at this location typically ranges from 0.8 to 1.2 m; however, it may experience a sudden increase during torrential rainfall due to Garaet’s role as a basin that is filled by rainwater runoff from the surrounding mountains. The climate is typically Mediterranean with a short wet period between October and February and a long dry season between March and September. This freshwater water body is located about 20 km from the Mediterranean Sea. It harbors a wide range of aquatic avifauna throughout the year [35]. It also hosts a marked floral diversity (mainly *Nymphaea alba*, *Typha angustipholia*, *Phragmites australis*, *Scirpus maritimus*, *S. lacustris*, *Iris pseudoacaurus*, and *Salvinia natans*). The land surrounding the wetland is used exclusively by local residents for agriculture of fruits such as watermelon, melon, and tomato [35].

### 2.2. Bird Counts

**Figure 1:**
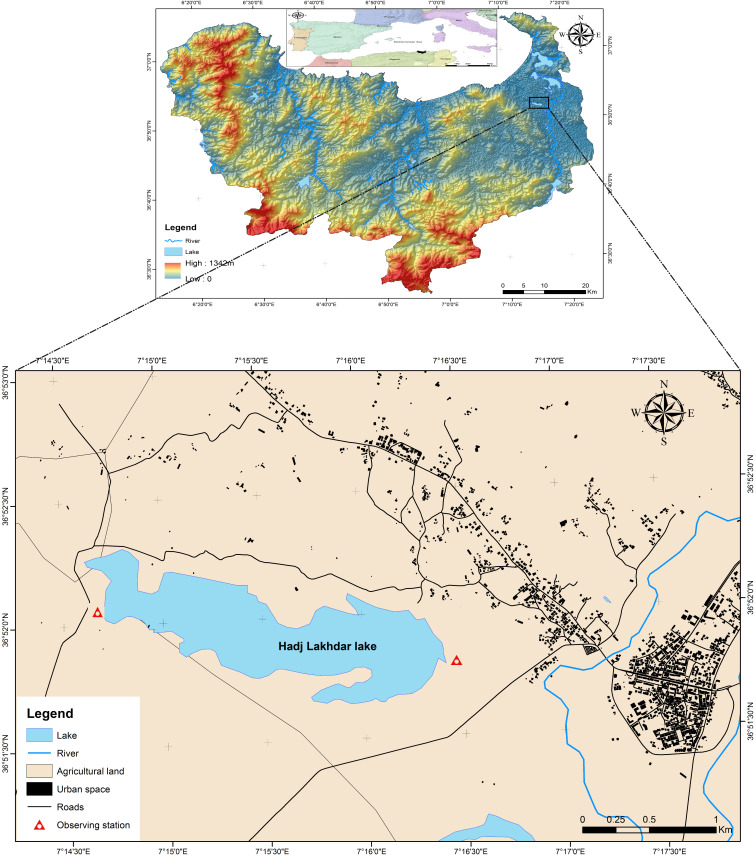
Map showing the study site at the North African Ramsar site, Garaet Hadj Tahar, during the period 2002–2019.

To assess the yearly fluctuations in the population size of four waterbird species during the wintering (September–February) and breeding seasons (March–August), we performed monthly counts of birds every year from 2002 to 2019 using a telescope (Konus 20 × 60). We used the same observation points consistently every year, which provided the best visibility and accessibility to the wetland (Figure 1). We believe that the population estimates should be representative of the entire wetland. To minimize observer bias, the sampling took place in the morning at 11:00–12:00 by one experienced observer trained by the same researcher. Every year, we collected 12 data points for each species, which we divided into two periods: wintering (September–February) and breeding seasons (March–August). Overall, we had a balanced dataset of 216 observations (18 years × 12 months) for each species (216 observations × 4 species = 864 observations). The bird estimates were carried out using Bibby, Jones [36]’s estimation method. This consists of counting the population size individually when the number of individuals per species was <200, or estimating visually the number of individuals when it either exceeds 200 or the birds are far away.

To estimate the number of active nests during the study period (2002 to 2019), trained researchers performed two prospections per week of each potential habitat type of the four studied species, starting from April to July. Since the wetland was relatively small, we were able to search the vegetation of all potential nesting sites using a kayak. Whenever a nest was found, it was marked with an individual code to avoid duplicate counts. We calculated the total number of nests for each species every year, resulting in a balanced dataset of 72 observations (18 years × 4 species).

### 2.3. Climatic Variables

To determine the change in climatic conditions in the wetland during the study period (2002–2019), we used historical monthly values of the average (Tm), minimum (Tmin), and maximum temperature (Tmax), as well as the annual precipitation (P) [37] and the Standardized Precipitation Evapotranspiration Index (SPEI) [38]. Temperature and precipitation data were obtained from WorldClim v2 (2.5 min spatial resolution) [37].

#### Relating Climate Variability and Population Dynamics

We calculated the average Tm, Tmin, Tmax, and SPEI, as well as the sum of P during specific time windows that vary in duration (1, 2, 3, …, and 12 months) and position (one-month shift). Thus, each time window has a duration (number of months) and a position (labeled by the last month of the window). The calculation of the rolling mean and sum of each time window was carried out using the roll_mean and roll_sum functions of the RcppRoll package [39]. Because the abundance of waterbirds could be influenced by climatic conditions of the focal and previous year [40], we carried out these time window calculations for both the focal and previous year. For instance, for the breeding season of 2015, we tested not only the time windows of 2015 (e.g., March 2015 [one month], February–March 2015 [two months], January–March 2015 [three months]), but also those of 2014 (e.g., March 2014, February–March 2014, January–March 2014). This resulted in 144 time windows for each climatic variable. To determine the best time window that correlates with the year-to-year fluctuations of bird abundance, we first ranked the absolute of the coefficient of correlation between bird abundance and the climate measure (Tm, Tmin, Tmax, P, and SPEI) of specific time windows, and selected the time window that had the highest absolute Spearman correlation coefficient (Table 1). We performed this analysis for each species for both the wintering and breeding seasons. We compared the correlation coefficient values of all climatic variables and used the best performing one for further modeling.

### 2.4. Vegetation Cover

Because the vegetation cover is an integral habitat feature for waterbirds [41,42], we calculated the yearly percentage of interior vegetation cover of the wetland (inside the marsh) during 2002–2019 based on the satellite free available data Landsat [43] with QGIS [44]. We extracted remote sensing images from different Landsat sensors, specifically Landsat 7 Enhanced Thematic Mapper Plus (ETM+) and Landsat 8 Operational Land Imager (OLI), and we used USGS Earth Explorer to download the images. The sensors had the same spatial resolution (30 m) and spectral ranges. We used spectral indices (Normalized Difference Vegetation Index (NDVI) and Normalized Difference Water Index (NDWI)) to assess the dynamics of vegetation cover during 2003–2019 [45]. Surface reflectance bands of Landsat 7 and 8 are used to compute NDVI as follows:

(1) $$DVI=\;\frac{NIR-RED}{NIR+RED}$$

where NIR and RED refer to the reflectance of Band 4 and Band 3 used in Landsat 7; Band 5 and Band 4 used in Landsat 8, respectively. NDVI values range between −1 and +1. NDVI > 0.2 was classified as vegetation.

### 2.5. Statistical Analysis

We carried out our statistical analysis with the R 4.2.2 software [46]. Values hereafter are mean ± SD.

Temporal trends of climate and vegetation

To assess the temporal pattern of climate and physical aspects of the wetland, we performed linear regressions with Tm, Tmin, Tmax, P, SPEI, and vegetation cover as response variables and year as an explanatory variable. We tested for the correlation of vegetation cover with Tm, Tmin, Tmax, P, and SPEI using Spearman’s correlation.

Temporal trends of bird abundance 

We tested for difference in the abundance of species using a likelihood ratio test that compares a generalized linear model with quasi-Poisson error distribution, including species as a predictor with a null model using lrtest function from the lmtest package. We used quasi-Poisson error distribution instead of Poisson because of the overdispersion detected using the function dispersion test from the AER package [47]. The temporal pattern of the number of individuals during the wintering (September–February) and breeding (March–August) seasons and the number of nests for all species were assessed using generalized models with quasi-Poisson error distribution. In addition, we also tested the quadratic relationship of year. To visualize the temporal trend of bird abundance, we fitted LOESS (locally estimated scatterplot smoothing) curves using the geom_smooth (method = ‘loess’) function of the ggplot2 package (v3.5.1) [48].

Determinants of bird abundance 

We used a generalized model with quasi-Poisson error to test whether bird counts (response variable) were correlated with the additive effect of the best climatic variable (determined with the best time window) and vegetation cover as explanatory variables. We checked for collinearity in our models using the vif (variance inflation factor) function of the car package [49] (vif in all models was <2, indicating a low level of multicollinearity among predictors).

## 3. Results

### 3.1. Environmental Changes

#### 3.1.1. Climate

**Figure 2:**
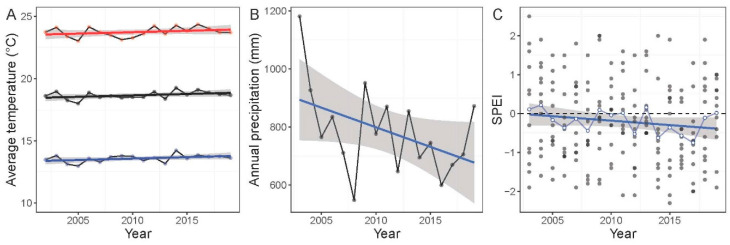
Temporal trends of temperatures (**A**), precipitation (**B**), and SPEI (**C**) at the North Africa Ramsar site Garaet Hadj Tahar during 2002–2019. (**A**) Annual average (black), minimum (blue), and maximum (red) temperature. (**B**) Annual precipitation. (**C**) Monthly values of SPEI are shown in gray (filled) points and annual average values are displayed in blue (open) points. Lines are linear regressions, and ribbons are standard errors.

Among all the climate variables checked for annual trend, only the precipitation from September to February had significantly declined, while the other variables did not significantly change linearly. The average annual temperature was 18.7 ± 0.3 °C, and it showed a slight but not significant increase during 2002–2019 (LM: t = 1.72, *p* = 0.10). Similarly, average minimum and maximum temperature did not show a significant temporal trend (LM: t = 1.72, *p* = 0.10; t = 1.29, *p* = 0.21, respectively) (Figure 2A). The average temperature of the four seasons did not show a significant trend (*p* = 0.40–0.70). Annual precipitation showed a marginal negative temporal trend (LM: t = −1.93, *p* = 0.07), with an average rate of decline of 13.5 mm yr^−1^ (Figure 2B). However, the sum of precipitation during the wet period (September–February) declined significantly, with an average rate of decline of 15.3 mm yr^−1^ (t = −2.20, *p* = 0.04). Annual precipitation was marginally significantly correlated with annual average temperature (r = −0.43, *p* = 0.08). Average annual SPEI showed a negative but not significant temporal trend (t = −1.60, *p* = 0.12) (Figure 2C). However, average SPEI during the wet period (September–February) showed a marginal temporal decline (t = −1.92, *p* = 0.07).

#### 3.1.2. Vegetation Cover

**Figure 3:**
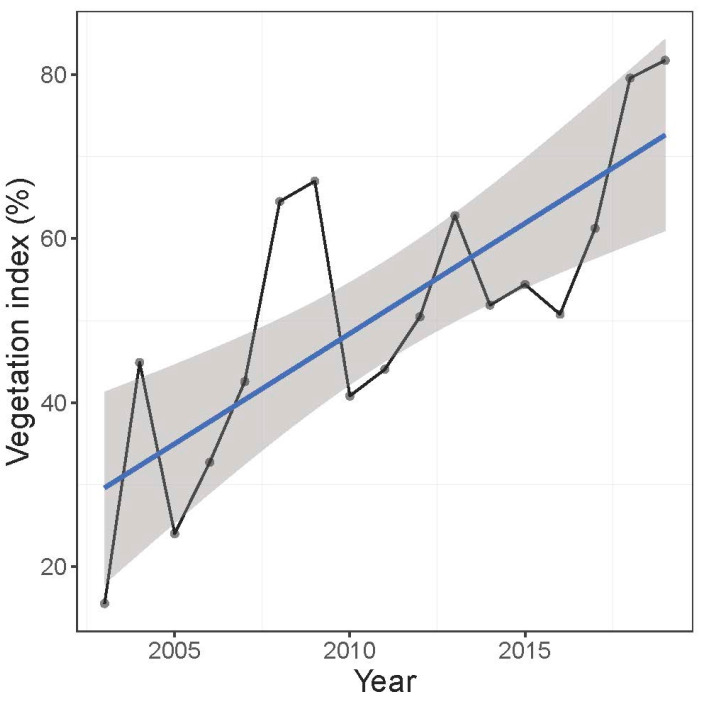
Temporal pattern of the vegetation cover of the North Africa Ramsar site, Garaet Hadj Tahar, during 2002–2019. Lines is linear regression, and ribbon is standard error.

There was a strong positive temporal trend of vegetation cover during 2002–2019 (LM: t = 4.55, *p* = 0.0004) (Figure 3). Vegetation cover increased on average by 2.7 ± 0.6% (±SE) per year. Vegetation cover was not significantly correlated with annual average temperature (Spearman correlation: r = 0.08, S = 792, *p* = 0.91), minimum temperature (r = 0.21, S = 639, *p* = 0.40), maximum temperature (r = −0.04, S = 856, *p* = 0.85), precipitation (r = −0.19, S = 974, *p* = 0.45), and SPEI (r = −0.05, *p* = 0.84).

### 3.2. Temporal Trends of Abundance

#### 3.2.1. Abundance During the Wintering Season

**Figure 4:**
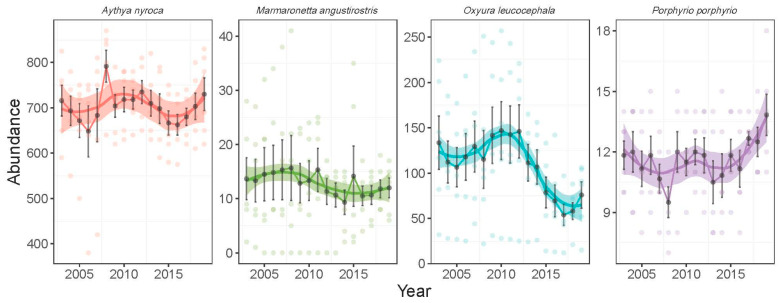
Temporal pattern of the abundance of four waterbird species during the wintering season in Gareat Hadj Tahar during 2002–2019. Wintering period is defined here as September–February (six months). Curves are LOESS regressions (locally estimated scatterplot smoothing) with standard error.

There was a significant difference in the abundance of species (likelihood ratio test: χ^2^ = 144,965, *p* < 0.0001) (Table 1). From the most to the least abundant species, we had *A. nyroca* (701.8 ± 82.9, range: 380–870), *O. leucocephala* (108.5 ± 62.3, range: 12–257), *M. angustirostris* (12.9 ± 8.8, range: 0–41), and *P. p. porphyrio* (11.6 ± 2.12, range: 7–18). The four species showed different temporal patterns of abundance during the wintering seasons from 2002 to 2019 (Figure 4). *A. nyroca* showed no clear temporal trend (GLM: z = −0.065, *p* = 0.948), although some year-to-year fluctuations were recorded. The minimum and maximum abundance for *A. nyroca* were recorded in September 2006 (380 individuals) and February 2008 (870 individuals), respectively. The wintering population of *M. angustirostris* did not show a significant trend (GLM: t = −1.38, *p* = 0.17), although estimates in recent years (14–16 individuals during 2003–2008) were slightly lower than those in earlier years (9–12 individuals during 2014–2019). *O. leucocephala* showed a quadratic trend (GLM: year, t = 3.05, *p* = 0.003; year^2^, t = 3.05, *p* = 0.002), where the peak average abundance was observed in 2010 followed by a marked decline in later years. Yearly average abundance of the first five years (2003–2007) was 106–134 individuals, the five last years (2015–2019) was 53–78 individuals, and the middle period (2008–2014) was 107–146 individuals. *P. p. porphyrio* showed a quadratic trend (GLM: year, t = −2.52, *p* = 0.01; year^2^, t = 2.52, *p* = 0.01), with a decline in early years (12 individuals in 2003 to 9 individuals in 2009), a plateau during the early 2010s (11–12 individuals during 2010–2014), and an increase in later years (12 individuals in 2015 to 14 individuals in 2019).

#### 3.2.2. Abundance During the Breeding Season

**Figure 5:**
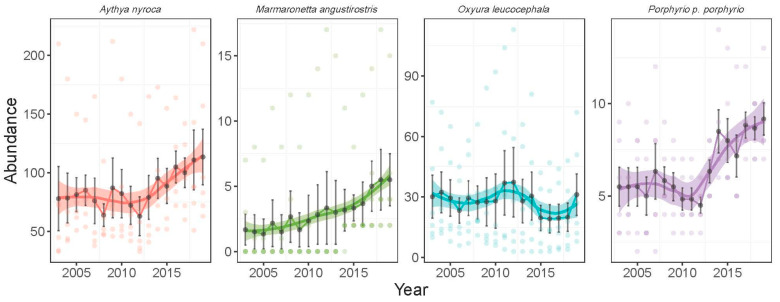
Temporal pattern of the abundance of four waterbird species during the breeding season in Gareat Hadj Tahar during 2002–2019. Breeding period is defined here as March–August (six months). Lines are LOESS regressions (locally estimated scatterplot smoothing) with standard error.

The apparent abundance of the four species during the breeding season differed significantly among species (likelihood ratio test: χ^2^ = 13,948, *p* < 0.0001) (Table 1). The average abundance of the four species from the most to the least abundant was 83.9 ± 44.9 (range: 33–222) individuals for *A. nyroca*, 27.6 ± 24.4 (3–113) individuals for *O. leucocephala*, 6.5 ± 2.6 (2–14) individuals for *P. p. porphyrio*, and 2.9 ± 4.3 (0–17) individuals for *M. angustirostris*. Apart from *O. leucocephala*, the other species showed an increasing temporal trend of abundance during the breeding seasons from 2003 to 2019 (Figure 5). *A. nyroca* showed a quadratic temporal trend (GLM: year, z = −7.40, *p* < 0.0001; year^2^, z = 7.40, *p* < 0.0001), with a marked increase in the last decade. The minimum abundance for *A. nyroca* was recorded in 2008 (64 ± 24 individuals) and the maximum was recorded in 2019 (114 ± 58 individuals). *M. angustirostris* abundance showed a positive trend (GLM: t = 2.79, *p* = 0.006). Yearly average abundances varied between 1.3 and 2.1 individuals during 2003–2007 and between 3.3 and 5.5 individuals between 2015 and 2019. The breeding population of *O. leucocephala* did not show a significant trend (GLM: t = −0.88, *p* = 0.38), although the number of individuals during 2015–2018 was slightly lower than previous years. *P. p. porphyrio* showed a similar quadratic trend as *A. nyroca* (GLM: year, z = −2.33, *p* = 0.02; year^2^, z = 2.34, *p* = 0.02), indicating a sharp increase in the last decade (4.5–6.3 individuals during 2003–2009 and 4.8–9.2 individuals during 2010–2019).

#### 3.2.3. Abundance of Nests

**Figure 6:**
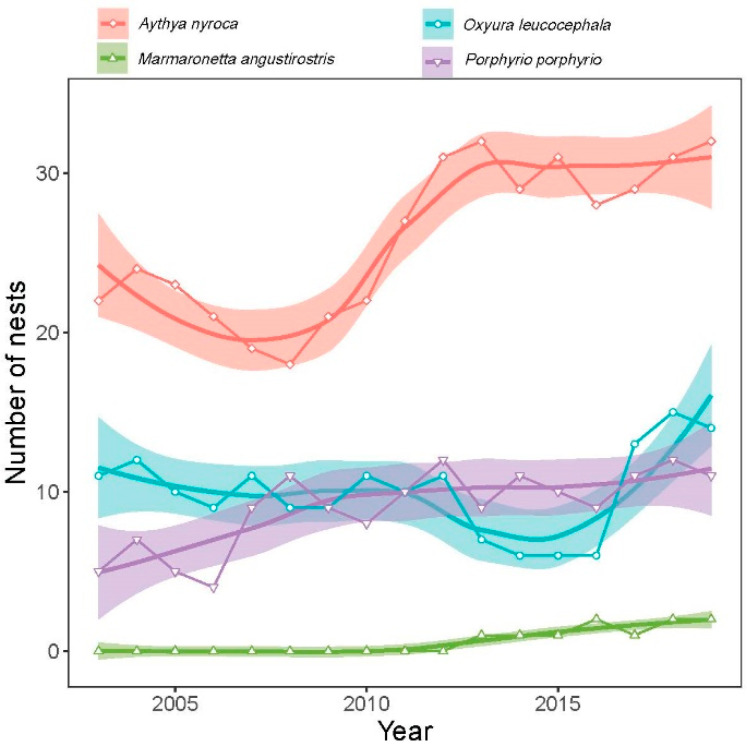
Temporal pattern of the number of nests of four waterbird species during the breeding season in Gareat Hadj Tahar during 2002–2019. Lines are LOESS regressions (locally estimated scatterplot smoothing) with standard error.

The number of nests recorded in the study sites during the study period differed significantly between species (likelihood ratio test: χ^2^ = 549.8, *p* < 0.0001) (Figure 6, Table 1). From the highest to the lowest number of nests, we had *A. nyroca* (25.9 ± 4.8, range: 18–32), *O. leucocephala* (10 ± 2.7, range: 6–25), *M. angustirostris* (9 ± 2.5, range: 4–12), and *P. p. porphyrio* (0.6 ± 0.8, range: 0–2). The number of nests had a positive temporal trend for *A. nyroca* (GLM: t = 4.9, *p* = 0.0001), *M. angustirostris* (GLM: t = 6.0, *p* < 0.0001), and *P. p. porphyrio* (GLM: t = 4.1, *p* = 0.001), but a moderate quadratic trend for *O. leucocephala* (GLM: year, z = −2.55, *p* = 0.02; year^2^, z = −2.55, *p* = 0.02). The number of *O. leucocephala* nests decline in the middle of 2010s (6 nests in 2014–2016) and increased in later years (13–15 nests in 2017–2019).

### 3.3. Correlation of Temporal Trends with Climate Change

The climatic variable that showed the highest correlation coefficients with the year-to-year fluctuations of the abundance of *O. leucocephala* during both the wintering (r = 0.51, *p* = 0.03) and breeding seasons (r = 0.72, *p* = 0.001) was SPEI (Table 1, Table S1). Wintering abundance of *A. nyroca* exhibited a higher correlation with precipitation (r = 0.58; *p* = 0.01), whereas breeding abundance was better correlated with Tmax (r = 0.61, *p* = 0.009). Both the wintering (r = 0.60, *p* = 0.01) and breeding population of *P. p. porphyrio* (r = 0.58, *p* = 0.01) were better correlated with Tmax. Tmean and SPEI showed higher correlations with the abundance of *M. angustirostris* during the wintering (r = −0.53, *p* = 0.03) and breeding seasons (r = −0.75, *p* = 0.001), respectively.

The best time window varied between species and seasons (Table 1). The duration of the time window varied between 1 and 12 months. The location (as labeled by the last month of the window) of the time windows varied between species, and involved different months from winter, spring, summer, and autumn of the previous year for both the wintering and breeding seasons (Table 1).

**Figure 7:**
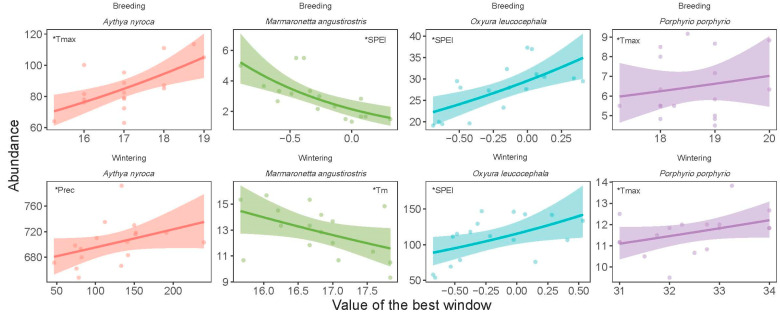
Change in the abundance of four waterbird species in Gareat Hadj Tahar during 2002–2019 with yearly values of the best time window for the wintering and breeding seasons. Lines are generalized regression with quasi-Poisson errors. Ribbons are standard error. The variable that best explains the change in abundance for each species and season is shown in each panel with *.

The increase in SPEI was associated with an increase in the abundance of the wintering (t = 2.28, *p* = 0.03) and breeding (t = 3.16, *p* = 0.007) population of *O. leucocephala* (Figure 7, Table 1), indicating that in wetter years, population size was larger. The population size of *A. nyroca* during the wintering season showed a marginally significant positive correlation with precipitation (GLM: t = 2.12, *p* = 0.10), whereas that during the breeding season exhibited a positive correlation with Tmax (t = 2.53, *p* = 0.02). An increase in Tmax was positively associated with an increase in population size of *P. p. porphyrio* during the wintering (t = 2.33, *p* = 0.03) and breeding seasons (t = 1.82, *p* = 0.09). The abundance of *M. angustirostris* during the wintering seasons was negatively correlated with Tm (t = −2.24, *p* = 0.04), whereas that of the breeding seasons was negatively correlated with SPEI (t = −3.63, *p* = 0.002).

Table 2 presents the species-specific statistics of the generalized model testing the additive effect of climate and vegetation on bird population size during the wintering and breeding seasons. Vegetation cover was positively correlated with the population size of *O. leucocephala* in the wintering seasons (t = 2.28, *p* = 0.03), *P. p. porphyrio* during the breeding seasons (t = 2.61, *p* = 0.02), and *M. angustirostris* during the breeding seasons (t = 4.01, *p* = 0.001).

## 4. Discussion

Recent studies have highlighted that waterbirds in arid regions such as North Africa are threatened by climate change and anthropogenic degradations [21,50,51], calling for more long-term studies to understand their contribution to population fluctuations. Our study assesses the temporal pattern of abundance of four threatened species in a wetland of international importance during the last two decades—a period characterized by severe climate warming, drought, and anthropogenic expansion across wetlands. We found that the temporal trend of waterbird abundance differed across species and seasons. Interestingly, the drought index performed better than Tm, Tmin, Tmax, or P in explaining the fluctuations in the population of the endangered *O. leucocephala* during the wintering and breeding seasons. Species exhibited different responses to climatic fluctuations, but the most endangered species (*O. leucocephala*) showed population declines in both wintering and breeding seasons in drier years. Moreover, the temporal increase in vegetation cover was positively correlated with the breeding population of *P. p. porphyrio* and *M. angustirostris*. We discuss below the implications of these results for the conservation of waterbirds in the region.

### 4.1. Vegetation Cover

We recorded an increase in the vegetation cover of the studied wetland during the last two decades. Studies have shown that various ecosystems worldwide have experienced an increase in vegetation cover [52,53,54]. In Egypt, vegetated areas of coastal wetlands in El-Burullus Lake increased by over 7% from 1990 to 2019 [55]. In Southern China, in the Dongting Lake basin, over 65% of the area showed an increasing trend in vegetation NDVI [56], whereas in the southeastern part of the country (Poyang Lake), the vegetation area increased by 15.5% of the lake area during 1987–2017 [57]. Similarly, in the Siberian lowland tundra (Russia), the percentage of sedge and *Sphagnum* vegetation increased by 10% and 22%, respectively, during 2010–2019 [58]. Therefore, the observed expansion of vegetation at the studied Ramsar site in Northeastern Algeria is a geographically widespread trend, suggesting that the drivers are cosmopolitan in nature [59]. It is likely that the recent rapid increase in vegetation cover is partly due to climate warming, promoting growth and expansion of aquatic plants within the wetland. In fact, the mechanisms underlying plant growth (respiration and photosynthesis) are highly sensitive to environmental temperature [60], and warming could lead to faster growth if the environmental temperature does not exceed the thermal optimum [61]. As shown in previous studies, macrophytes of shallow wetlands like the studied site can emerge earlier and become more productive when temperatures increase [62]. Furthermore, Garaet Hadj Tahar is surrounded by many farmlands, thus nutrient runoff (e.g., nitrogen and phosphorus) could reach the wetland and change vegetation structure [59,63]. Further studies need to unravel the underlying causes of this increase in vegetation density in wetlands and their relative contribution.

### 4.2. Wintering Population

The wintering population of different waterbird species showed distinct temporal trends in Garaet Hadj Tahar. Notably, *O. leucocephala* showed a marked decreasing pattern in later years, from 107–146 individuals (2008–2014) to 53–78 individuals (2015–2019). In winter, *O. leucocephala* prefers to occupy open areas within lakes and marshes [64] to perform various activities, including feeding and resting [65]. We hypothesized that an increase in vegetation density might influence directly or indirectly the wintering population size of *O. leucocephala*, such that an increase in vegetation density could shrink the open area for the wetland, reducing the carrying capacity of the species. However, our analysis showed the opposite pattern (positive correlation between population size and vegetation cover). A change in vegetation structure could be associated with a shift in the composition of essential food resources for the species (macroinvertebrates and plant seeds) [66], which in turn positively influence the wintering population size. Additionally, nutrient runoff from nearby agricultural activities could have contributed to increased vegetation growth and primary productivity in the wetland, indirectly benefiting waterbirds. A previous study has shown that nutrient enrichment can positively affect wetland productivity and support higher waterbird densities by enhancing food availability [67].

The abundance of *A. nyroca* fluctuated from one year to another at around 700 individuals, which is higher than many estimates reported for the species in other sites in the region (e.g., Lac des Oiseaux, Boussedra, Lake Mellah) [68,69]. Unlike our study site, the wintering population in Morocco has shown a remarkable increase during the last few decades [70], suggesting that different North African wetlands have undergone various changes in environmental conditions. Furthermore, *Porphyrio p. porphyrio* showed a small population, with a slight increase in size in the past decade. Studies have mainly reported small numbers in some wetlands in North Africa [71,72]; thus, our records are typical in the region. The increase in population size might be due to the increase in the frequency of breeding in the sites, as indicated by our records of larger breeding populations and higher number of nests. The wintering population of *M. angustirostris* did not show a clear temporal trend. This waterbird has regularly been observed in small numbers in the study site, probably because the local climate is not as optimal as the species prefers (semiarid and arid). In fact, the population size of the species in the south where the climate is drier is remarkably larger than that recorded in our study [73,74]. Such a geographic pattern was also observed in Morocco [75]. Although the site hosts only a small population, it is among the rare sites reported in the North of the country where the species is found all year round. Considering the rapid change in climatic conditions in the North, which are predicted to become hotter and drier in the future [21], the site could exhibit a gradual increase in the wintering population size of *M. angustirostris* in the near future as a result of range shift in emigration northwards [76].

Despite the preference of vegetated wetlands [77,78,79], we did not find a correlation between vegetation cover and wintering population size of *A. nyroca*, *Porphyrio p. porphyrio*, and *M. angustirostris*. This suggests that the environmental changes driven by the shift in vegetation structure, as well as the associated changes in the habitat and food requirements of the species did not influence the demographic parameters. Further studies are needed to disentangle the contribution of changes in vegetation cover on waterbird behavior and population dynamics.

### 4.3. Breeding Population

The abundance of *O. leucocephala* during the breeding season showed a slight decrease in the last decade, particularly during 2013–2016, associated with a reduction in the number of recorded nests. However, the breeding population seemed to have slightly recovered later. The abundances recorded for this endangered species were smaller than those reported in other Northeast Algerian wetlands, particularly in Lake Tonga [80] and Lake Boussedra [81] (Northeast Algeria). *A. nyroca* was the most abundant breeding species among the four surveyed species on the site, showing a marked increase in breeding population and number of active nests. The abundances of *O. leucocephala* and *A. nyroca* during the reproductive season were not correlated with vegetation cover, suggesting that the species have not benefitted from the increase in the availability of vegetation, as suggested in other previous studies [79,82], including those suggesting higher reproductive success of nests in areas with higher (often denser) vegetation cover [83].

Despite the small size of the breeding population of *M. angustirostris*, it is one of the rarest northern Ramsar sites with a confirmed reproduction. It is likely that the recent increase in the small local breeding population is due to an increase in the birth and recruitment rate because of the recorded increase in the number of nests. Similarly, *P. p. porphyrio* showed an increase in the breeding population, particularly in the number of nests recorded at the site.

The breeding population size of *M. angustirostris* and *P. p. porphyrio* showed a positive correlation with vegetation cover, suggesting that the environmental changes that occurred on the site benefited the species. This preference for vegetated wetlands corroborates with previous studies on these species in the Mediterranean region. A study in Morocco showed through habitat-based statistical models that the occurrence probability of *M. angustirostris* at Moroccan wetlands was positively associated with the number of emergent vegetation species [78]. In the Iberian Peninsula, *P. p. porphyrio* increased in numbers and expanded its range relatively rapidly after a severe population decline during the first half of the 20th century [84,85]. In Florida, the species was introduced in the 1990s and shortly increased its numbers and range [86]. Thus, under more favorable environmental conditions, the demographic parameters of the species are optimized to swiftly grow in population size and rapidly establish colonies.

### 4.4. Climate Change Influence

The drought index (SPEI) performed better than temperature and precipitation in predicting the number of individuals of *O. leucocephala* in both the wintering and breeding season. This better performance is probably due to the integration of evapotranspiration and precipitation in the same metric, which likely gives a better representation of the ecological requirement of the species than temperature and precipitation alone. The negative correlation between drought intensity and population size suggests that the habitat requirements of the species are not met in drier years. In fact, water level, water surface, prey availability, and carrying capacity of wetlands are often lower in drier years [87,88,89], which probably incite a fraction of the population to look for alternative sites where foraging success and breeding probability is higher [90,91]. As divers, *O. leucocephala* could be influenced by a reduction in water levels, potentially affecting their feeding behavior and altering the composition of macroinvertebrates and plant seeds, which represent the main components of the species diet [68]. Studies on Australian waterbirds have highlighted similarly complex relationships between climatic conditions and waterbird abundance, showing that broader-scale hydrological patterns (e.g., streamflow, regional rainfall) influence movement dynamics, even in areas with permanent water bodies; for instance, long-term studies in Australia have found that certain waterbirds respond not only to local wetland conditions but also to large-scale climatic drivers like the Southern Oscillation Index (SOI) and streamflow in distant regions [92]. These findings suggest that the response of *O. leucocephala* to climate variability may involve more than just local water retention and could reflect broader hydrological and ecological processes.

The other waterbird species showed interspecific difference in their response to drought and warming, a pattern that is consistent with other studies [18,93]. For instance, drought probably had a positive effect on *M. angustirostris* during the breeding season because the species showed larger population sizes in drier years. This is probably due to the preference for drier conditions [73], which is consistent with the geographic distribution of the species. Higher temperature extremes seemed to have benefitted *A. nyroca* and *P. p. porphyrio*. This variation in waterbird responses could be due to the changes in food resources and habitat suitability, benefitting some but impeding other species [94]. All three waterbirds that benefited from warmer and dry conditions feed (at least partially) on shallow waters, which might have increased in frequency in drier years. In recent years, studies have reported these species in various urban and artificial sites in Northeast Algeria, particularly in the Northeast [68,95], suggesting a local population growth and expansion. For instance, Boussedra Marsh, which was recently designated as an Important Bird Area (IBA) [72], harbors a relatively important wintering (146–258 individuals) and resident (<50 individuals) population of *A. nyroca* [68]. Such spatial shifts in warming and drought periods suggest that the species have a broad ecological niche, which allows them to withstand stressful conditions [96]. Unfortunately, in the absence of data about population dynamics in a large network of wetlands, and a lack of information about the climate-driven changes in biotic impacts (e.g., interspecific competition, predation, food availability) [97] as well as their seasonal patterns [98], it is difficult to explain demographic drivers of the observed changes in population size. Research has demonstrated that variations in environmental factors, such as temperature and precipitation, directly influence the abundance and diversity of waterbird populations. For example, a study conducted at Yamzho Yumco Lake revealed notable shifts in waterfowl communities between 2015 and 2021, underscoring the disruptive effects of climate-driven environmental changes on population dynamics [99]. The impact of winter weather is particularly significant; in southern Europe, drier winters can degrade wetland quality and reduce habitat availability for wintering waterbirds, whereas in northern regions, increased precipitation may create more favorable breeding conditions [100]. This indicates that climate change affects different regions in distinct ways, potentially forcing species to shift from their traditional habitats. Furthermore, the functional diversity of waterbirds varies across geographic and environmental gradients and is strongly linked to climate conditions. Temperature and precipitation patterns shape wetland habitat availability and productivity, directly influencing species richness and community composition [101,102]. These findings emphasize the complex interactions between large-scale climatic factors and localized ecological conditions. Although waterbirds exhibit some resilience to environmental change, research suggests a delayed response in population dynamics to shifting climate patterns. As global temperatures continue to rise, waterbird communities may struggle to adapt at the same rate, leading to a phenomenon known as “climatic debt”, which could pose long-term risks to their sustainability [103]. While habitat conservation efforts can help mitigate these negative effects, effective strategies must account for the ongoing and rapid pace of climate change [104]. In addition, waterbirds play a vital role in coastal and freshwater wetlands, contributing significantly to nutrient cycling. Their waste products, which contain high concentrations of nitrogen and phosphorus, can substantially influence the physicochemical characteristics of water bodies and enhance ecosystem productivity [105,106]. For instance, the deposition of guano by waterbirds can increase total phosphorus and nitrogen levels, effectively fertilizing aquatic vegetation and promoting plant growth [107]. This nutrient enrichment supports the aquatic food web and strengthens the overall ecological integrity of wetlands. Additionally, waterbirds contribute to seed dispersal by transporting plant propagules through digestion and excretion, aiding in the spread of various plant species across different habitats. The diversity of waterbird species, coupled with their distinct feeding behaviors, influences the movement and distribution of both aquatic and terrestrial plants within wetland ecosystems [108,109]. Moreover, the seasonal movements of waterbirds often coincide with plant life cycles, fostering a recurring ecological interaction that enhances wetland stability and resilience [110,111].

Our results highlighted that Tmax performed better in predicting the wintering abundance of *A. nyroca* and *P. p. porphyrio*, which highlights the importance of temperature extremes and the limitations of the average temperature in explaining the population dynamics of waterbirds. Recent studies have highlighted the importance of temperature extremes in studying the impact of climate change on biodiversity [112,113]. Waterbirds were shown to be sensitive to winter heatwaves, reducing their occurrence. During heatwaves, seabirds were observed to reduce nest attendance, which reduces reproductive success [114]. In addition to potential mortality due to climatic extremes, waterbirds can withstand thermal extremes by employing a variety of strategies. First, birds could thermoregulate if they have the necessary energetic reserves [115]. Second, birds could occupy optimal microhabitats as refugia to temporarily avoid extreme thermal events [116]. Third, waterbirds could leave the site and look for an alternative wetland that offers better environmental conditions [117]. Further studies are needed to disentangle the behavioral strategies that waterbirds adopt to avoid extreme climatic events in our study site.

### 4.5. Limitations

Our study still carries some limitations that we highlight here. Despite the large efforts undertaken throughout these two decades, our data are still limited to a single site, which limits our ability to generalize our results across broader areas. Thus, we acknowledge that the documented climate–abundance relationships may vary across space. Although our regression analyses revealed statistically significant associations between population dynamics and environmental variables, we interpreted these results cautiously and emphasized only those relationships that are biologically plausible and supported by the existing literature on species’ responses to environmental change. Nevertheless, this study remains one of the first long-term assessments of threatened birds in an Algerian wetland of conservation priority.

## 5. Conclusions

The North African region is not only an integral part of the Mediterranean hotspot of biodiversity [94], but also a host for a large number of Ramsar sites and IBAs that harbor a significant portion of the global population of threatened species [21]. Our study investigated the year-to-year fluctuations of four species of waterbirds and assessed the potential correlation of climate change and variation in vegetation cover with population fluctuations. Our results suggest that all four species were influenced by climate change, but this effect depended on the species and the season. However, the most endangered species (*O. leucocephala*) was the one that showed population decline in drier years. It is important to establish a conservation plan that increases the resilience of wetlands under stressful climatic conditions (e.g., reducing wetland use for irrigation, and building artificial wetlands) to protect this species. The presented data are one of the rarest long-term observation schemes in a Ramsar site in North Africa. Nevertheless, a collaborative network of observers is needed to assess population trends in multiple sites, especially those of international importance. Such an effort would provide critical information about the drivers influencing population trends and would inform decision makers to improve the conservation status of waterbirds under global change.

## Figures and Tables

**Table 1:** Best time window of the population dynamics of four waterbird species in a North African Ramsar site during the wintering and breeding seasons. The correlation coefficient results from spearman correlations. Window location is labeled with the last month of the window (e.g., 12 = December).

Season	Species	Corr.Coef	*p*	Variable	Window Duration (Month)	Window Location
Wintering	*Aythya nyroca*	0.580	0.015	Prec	2	10
	*Marmaronetta angustirostris*	−0.530	0.029	Tmean	3	12
	*Oxyura leucocephala*	0.517	0.036	SPEI	12	4
	*Porphyrio p. porphyrio*	0.599	0.011	Tmax	2	8
Breeding	*Aythya nyroca*	0.609	0.009	Tmax	1	12
	*Marmaronetta angustirostris*	−0.747	0.001	SPEI	8	7
	*Oxyura leucocephala*	0.719	0.001	SPEI	12	3
	*Porphyrio p. porphyrio*	0.584	0.014	Tmax	12	1

**Table 2:** Summary statistics of the generalized linear model with quasi-Poisson error distribution of the determinants of population fluctuation of four waterbird species in North Africa. (LCI and UCI: lower and upper confidence intervals, P: precipitation, Tmax: maximum temperature, SPEI: Standardized Precipitation Evapotranspiration Index).

Species	Season	Term	Estimate	LCI	UCI	Std. Error	Statistic	*p*
*Aythya nyroca*	Wintering	Intercept	6.505	6.440	6.560	0.031	211.859	<0.0001
		Climate [P]	0.0001	−0.0001	0.001	0.0001	1.713	0.107
	Breeding	Intercept	2.812	1.710	3.910	0.561	5.016	0.0002
		Climate [Tmax]	0.088	0.020	0.156	0.035	2.532	0.024
		Vegetation	0.003	−0.001	0.007	0.002	1.288	0.218
*Oxyura leucocephala*	Wintering	Intercept	4.748	4.610	4.880	0.067	70.855	<0.0001
		Climate [SPEI]	0.387	0.053	0.718	0.170	2.282	0.037
	Breeding	Intercept	3.323	3.070	3.570	0.128	25.981	<0.0001
		Climate [SPEI]	0.446	0.170	0.723	0.141	3.164	0.007
		Vegetation	0.001	−0.004	0.006	0.003	0.539	0.598
*Porphyrio porphyrio*	Wintering	Intercept	0.564	−1.020	2.160	0.812	0.695	0.498
		Climate [Tmax]	0.058	0.009	0.107	0.025	2.329	0.034
	Breeding	Intercept	−3.428	−8.660	1.790	2.665	−1.286	0.219
		Climate [Tmax]	0.207	−0.015	0.430	0.114	1.824	0.090
		Vegetation	0.007	0.002	0.013	0.003	2.611	0.021
*Marmaronetta angustirostris*	Wintering	Intercept	4.488	2.940	6.030	0.789	5.691	0.0001
		Climate [Tm]	−0.105	−0.196	−0.013	0.047	−2.246	0.041
		Vegetation	−0.003	−0.007	0.000	0.002	−1.918	0.076
	Breeding	Intercept	0.070	−0.330	0.455	0.200	0.349	0.732
		Climate [SPEI]	−0.683	−1.050	−0.315	0.188	−3.632	0.003
		Vegetation	0.015	0.007	0.022	0.004	4.016	0.001

When vegetation is not present in the model, it means that its inclusion in the model influenced the significance of the climatic independent variable.

## Data Availability

The datasets used and/or analyzed during the current study are available from the corresponding author on reasonable request.

## Supplementary Materials

The following supporting information can be downloaded at: https://www.mdpi.com/article/10.3390/life15060892/s1, Table S1: Best time window for each climate variable for the population dynamics of four species in a North African Ramsar site during the wintering and breeding season.
