# Supplementary material for: Threatened Birds in a Changing Mediterranean Wetland: Long-Term Trends and Climate-Driven Threats
Source: Life (Basel). 2025 May 31;15(6):892. doi: 10.3390/life15060892 (PMC12194160; doi:10.3390/life15060892)
Supplement: Supplementary file 1 [file life-15-00892-s001.zip › life-3652327-supplementary.pdf]

life

MDPI

Supplementary material

Threatened Birds in a Changing Mediterranean Wetland: Long-Term Trends and Climate-Driven Threats

Imane Bouregbi [1], Zinette Bensakhri [2], Rabah Zebsa [2,*], Abdelheq Zouaimia [2], Soufyane Bensouilah [3], Oualid Bouteraa [4], Rassim Khelifa [5], Mohamed Laid Ouakid [1], Hayat Mahdjoub [5] and Moussa Houhamdi [2]

[1] Department of Biology, Faculty of Sciences, University Badji Mokhtar, B.P. 12, Sidi-Ammar, Annaba 23000, Algeria; bouregbi.imane@gmail.com (I.B.); ouakidmomo@outlook.fr (M.L.O.)
[2] Laboratoire Biologie, Eau & Environnement (LBEE), Faculty of SNV-STU, University of 8 May 1945 Guelma BP 4010, Guelma 24000, Algeria; bensakhri.zinette@univ-guelma.dz (Z.B.); zouaimia.abdelheq@gmail.com (A.Z.); houhamdi.moussa@univ-guelma.dz (M.H.)
[3] Department of Biology, Faculty of Sciences, Amar Telidji Laghouat University, Laghouat 03000, Algeria; soufyaneben@hotmail.com
[4] Laboratory of Geology and Environment (LGE), University of Constantine 1, Constantine 025000, Algeria; oualid.bouteraa25@gmail.com
[5] Biology Department, Concordia University, 7141 Sherbrooke St. W., Montreal, QC H4B 1R6, Canada; rassim.khelifa@concordia.ca (R.K.), hayatmahdjoub@gmail.com (H.M.)
* Correspondence: zebsa.rabah@univ-guelma.dz

Table S1. Best time window for each climate variable for the population dynamics of four species in a North African Ramsar site during the wintering and breeding season. The correlation coefficient results from spearman correlations. Window location is labeled with the last month of the window (e.g., 12=December).

Season	Species	Variable	Window duration (month)	Window location	Corr.coef	P
**Breeding**	*Aythya nyroca*	Tmax	1	12	0.609	0.009
	Aythya nyroca	Tmean	1	12	0.527	0.030
	Aythya nyroca	Tmin	12	6	0.446	0.072
	Aythya nyroca	SPEI	1	7	-0.436	0.080
	Aythya nyroca	Prec	8	9	-0.314	0.220
	Marmaronetta angustirostris	SPEI	8	7	-0.747	0.001
	Marmaronetta angustirostris	Tmean	1	9	0.634	0.006
	Marmaronetta angustirostris	Prec	7	6	-0.624	0.007
	Marmaronetta angustirostris	Tmin	1	9	0.602	0.011
	Marmaronetta angustirostris	Tmax	12	12	0.512	0.036
	Oxyura leucocephala	SPEI	12	3	0.719	0.001
	Oxyura leucocephala	Tmin	3	5	-0.696	0.002
	Oxyura leucocephala	Tmean	3	5	-0.682	0.003
	Oxyura leucocephala	Prec	12	3	0.650	0.005
	Oxyura leucocephala	Tmax	3	5	-0.542	0.025
	Porphyrio porphyrio	Tmax	12	1	0.584	0.014
	Porphyrio porphyrio	Tmin	10	6	0.583	0.014
	Porphyrio porphyrio	Tmean	7	3	0.570	0.017
	Porphyrio porphyrio	SPEI	6	2	-0.528	0.029
	Porphyrio porphyrio	Prec	11	2	-0.477	0.053
**Wintering**	*Aythya nyroca*	Prec	2	10	0.580	0.015
	Aythya nyroca	Tmax	10	5	-0.525	0.030
	Aythya nyroca	SPEI	2	10	0.520	0.033
	Aythya nyroca	Tmin	2	8	0.501	0.041
	Aythya nyroca	Tmean	9	5	-0.490	0.046
	Marmaronetta angustirostris	Tmean	3	12	-0.530	0.029
	Marmaronetta angustirostris	Tmax	4	12	-0.522	0.032
	Marmaronetta angustirostris	Prec	11	10	0.495	0.043
	Marmaronetta angustirostris	SPEI	7	6	0.489	0.047
	Marmaronetta angustirostris	Tmin	12	12	-0.484	0.049
	Oxyura leucocephala	SPEI	12	4	0.517	0.036
	Oxyura leucocephala	Prec	1	4	0.478	0.054
	Oxyura leucocephala	Tmax	12	1	-0.460	0.063
	Oxyura leucocephala	Tmean	11	5	-0.427	0.088
	Oxyura leucocephala	Tmin	10	6	-0.370	0.144
	Porphyrio porphyrio	Tmax	2	8	0.599	0.011
	Porphyrio porphyrio	Tmean	2	8	0.507	0.038

	Porphyrio porphyrio	Prec	11	11	0.479	0.052
	Porphyrio porphyrio	Tmin	2	7	0.457	0.065
	Porphyrio porphyrio	SPEI	1	7	-0.435	0.081
